# Growth mechanism of nano-plates structured SnS films on different substrates in glancing angle deposition method

**DOI:** 10.1038/s41598-022-22965-9

**Published:** 2022-10-26

**Authors:** M. R. Sazideh, M. H. Ehsani, M. M. Shahidi, H. Rezagholipour Dizaji

**Affiliations:** Faculty of Physics, Semnan University, Semnan, 35195-363 Iran

**Keywords:** Surfaces, interfaces and thin films, Condensed-matter physics

## Abstract

In this work, Tin (II) sulfide films have been deposited on glass, Indium Tin Oxide, and Fluorinated Tin Oxide substrates at the deposition angles of 0º, 65º, and 85º using Physical Vapor Deposition method equipped with Glancing Angle Deposition technique. Based on the results obtained from the X-ray diffraction technique, the crystalline structure of substrates and the angle of depositions along with their effects on the structure of SnS nano-plates have been investigated. Using Raman analysis, the phonons lifetime of the samples was found to change with the type of substrate and the employed deposition angle. Based Energy-dispersive X-ray spectroscopy analysis, the atomic ratio of Sn to S was observed to change with the change of deposition angle, substrate type and variation the diameter of nano-plates. This phenomenon resulted the formation of the second phase of Sn_2_S_3_ which was confirmed by Raman and X-ray diffraction patterns. The nano-sheets-like growth of all the samples has been confirmed using Felid Emission Scanning Electron Microscopy analysis. For further morphological studies, the Atomic Force Microscopy analysis has been applied, by which the direct relation between the substrate roughness and the final structure of the samples has been observed. The relation between the substrate roughness and the deposition angle in the growth process of SnS nano-sheets has been explained.

## Introduction

Thin films are an indispensable part of industrial applications in microelectronics, optoelectronics, detectors, and sensors, which have received great attention in recent decades^1^. As reported, the surface morphology of thin films controls the physical and chemical properties of the deposited materials. In this regard, the deposition process is found to have a great significant effect^1^. For example, 3-D control of the surface of materials has been found probable using Glancing Angle Deposition (GLAD) technique. This technique is capable of producing structures with regular patterns^2^. In the GLAD technique, a material flux with a high melting point reaches the surface substrate at a specified angle (α) with respect to the perpendicular axis of the source surface. Low-mobility ad-atoms cause the kinetic limitations such as geometrical confinements and atomic shadows, leading to the formation of a variety of porous columnar microstructures. Therefore, the GLAD technique is capable of producing nano-sheets with complicated structures. Two phenomena; shadowing and re-emission effects are reported to be the reason for the film surface roughness increment^3^. The roughness has a crucial role in the growth process of nano-sheets which stems from the structure and morphology of the seeds of the utilized substrates. Recently, tin (II) sulfide (SnS) has received a lot of attention due to its semiconductor, optical, and electrical properties. This semiconductor belongs to the group of layered semiconductors that have orthomorphic structure, where the Sn and S atoms are tightly bound together in one layer, and the layers are bonded by weak Van der Waals forces^4^. More importantly, for SnS, depending on the concentration of tin, both p- and n-type conductions have been reported^5^. The SnS thin films have a direct bandgap (1.32–1.5 eV)^6^ and an indirect bandgap (1–1.3 eV)^7^. This phenomenon suggests that SnS has electrical and optical features suitable for a wide variety of applications such as photovoltaic devices, solid-state lubricants, near-infrared detectors, lithium-ion batteries, and sensors^8^. This sustainability has the advantage that the constituent elements are abundant in nature and do not pose a threat to health and the environment. In addition, tin sulfides have different phases such as SnS, Sn_2_S_3_, Sn_3_S_4_, and SnS_2_^9^. The present investigation is a report on the growth mechanism of SnS nano-plates deposited on different substrate types by Glancing Angle Deposition technique to find what parameters can have direct effect on the growth mechanism. Also, the induced optical properties and the factors affecting it were investigated.

## Experimental details

SnS thin films have been deposited on glass, indium tin oxide (ITO), and Fluorinated Tin Oxide (FTO) substrates at the deposition angles of 0°, 65°, and 85° with respect to the source normal using Physical Vapor Deposition (PVD) technique (Hind-HIVAC Model 15FC). These processes have been carried out at room temperature under the chamber pressure of 10^–6^ mbar. More details have been reported elsewhere^10,11^. Subsequently, the as-prepared samples have been systematically studied by X-ray diffractometer (XRD) (ADVANCE–D8 model) with Cukα radiation source in the scanning range of 10°–80°, Field Emission Scanning Electron Microscope (FESEM) (MIRA3 TESCAN), Atomic Force Microscope (AFM) (Nanotechnology GmbH DS 95 Series), and Raman (URaman-532-Ci) technique and finally Energy-dispersive X-ray spectroscopy (EDAX) (VEGA\\TESCAN-LMU). To estimate the porosity and average roughness in the images of AFM, program ImageJ application (National Institute of Mental Health, Bethesta, Maryland, USA) was used to convert AFM images to binary and calculate the height of nano-sheets.

### XRD analysis

**Figure 1:**
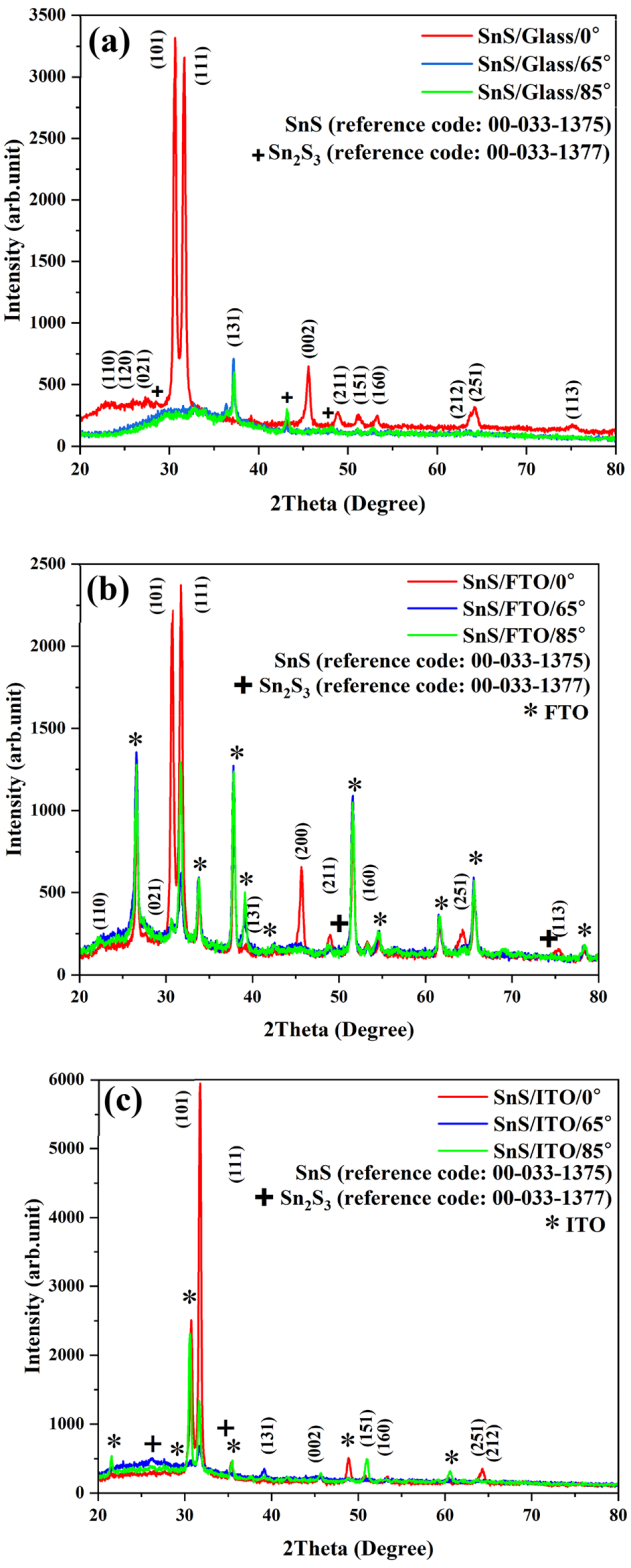
XRD patterns of (**a**) SnS. Glass, (**b**) SnS. FTO, (**c**) SnS. ITO, at different angle of deposition.

XRD spectra of tin sulfide layered samples on glass substrates, ITO and FTO are shown in Fig. 1 at the deposition angles 0°, 65°, and 85°. As it can be seen from the sample spectra, the predominant structure is orthorhombic and the predominant phase is SnS with the standard code JCPDS card # 00-03-1775. One of the features of the GLAD layering method is the amorphousness of the samples by increasing the layering angle, which is the existing feature of forming the second phase of the material (Sn_2_S_3_) with the standard code JCPDS card # 00–033-1377. Atoms tend to grow in directions that require the least amount of energy during deposition^12^. It is observed for preference from (101) to (111) in the angle of the non-zero address layer, other researchers have also observed this change^12^.

**Figure 2:**
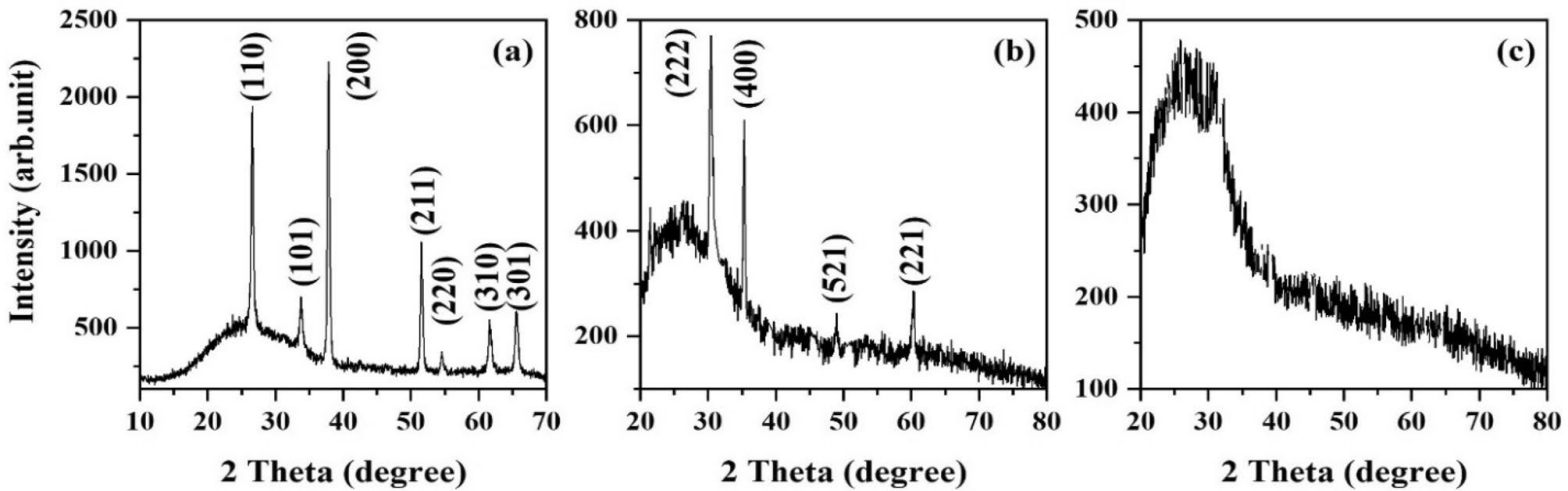
XRD patterns of (**a**) FTO, (**b**) ITO, (**c**) glass substrates.

One effective factor in thin film deposition of a material is the structure of the substrate. In this regard, for investigating the substrate effect, the ITO, FTO, and glass substrates were subjected to XRD analysis. Figure 2, shows the results of the same. ITO has a cubic structure with the standard card of JCPDS card # 00-044-1087, FTO has a tetragonal structure with JCPDS card # 01-077-0452 standard card, and glass substrate has amorphous nature without any special geometrical structure. As a result, using a substrate other than glass, led to producing a film having strain, despite the employed deposition angle.

This lattice strain ($\varepsilon$) can be obtained as following^13^:

1 $$\varepsilon =\frac{\beta\, cos\,cos \left(\theta \right) }{4}$$

The lattice strain has an important role in the structural and morphological properties of a film. As known, although all the orthorhombic, tetragonal, and cubic structures are orthogonal, the preferred crystalline orientation may be different. According to the XRD pattern, the emergence of lattice strain is a result of the changes in c orientation. Besides that, the film strain depends on other factors such as the substrate crystalline nature, change in the orientation of the preferred planes of the substrate, and the deposition method^5,6^.

It has been reported that the preferred planes at low 2θ values have less strain on the main structure, since they need low energy for growing^11^. The highest and the lowest values, respectively, belong to FTO and ITO substrates. With the increase of the deposition angle, the structure of the sample becomes amorphous, leading to an increase of lattice strain. The calculated lattice strain for the preferred orientation has been presented in Table 1.

**Table 1:** Different structural and morphological parameters of samples.

Samples	Porosity (%) (FESEM)	Porosity (%) (AFM)	β(^ο^)	Lattice strain		Diameter of nano-sheet (nm)
SnS.FTO.0^o^	0	27	0	(101)	0.686	19.23
				(111)	0.647	
SnS.FTO.65^o^	38	36	46.43	(101)	–	22.02
				(111)	0.660	
SnS.FTO.85^o^	51	55	54.54	(101)	−	24.23
				(111)	1.192	
SnS.glass.0^o^	0	21	0	(101)	0.603	13.19
				(111)	0.629	
SnS.glass.65^o^	31	32	43.32	(101)	–	16.19
				(111)	0.633	
SnS.glass.85^o^	44	47	51.61	(101)	–	17.84
				(111)	0.850	
SnS.ITO.0^o^	0	29	0	(101)	0.604	17.50
				(111)	0.491	
SnS.ITO.65^o^	35	39	45.33	(101)	–	19.10
				(111)	0.465	
SnS.ITO.85^o^	39	43	52.12	(101)	–	22.63
				(111)	0.860	
FTO Substrate				1.073		
ITO Substrate				0.522		

### RAMAN analysis

**Figure 3:**
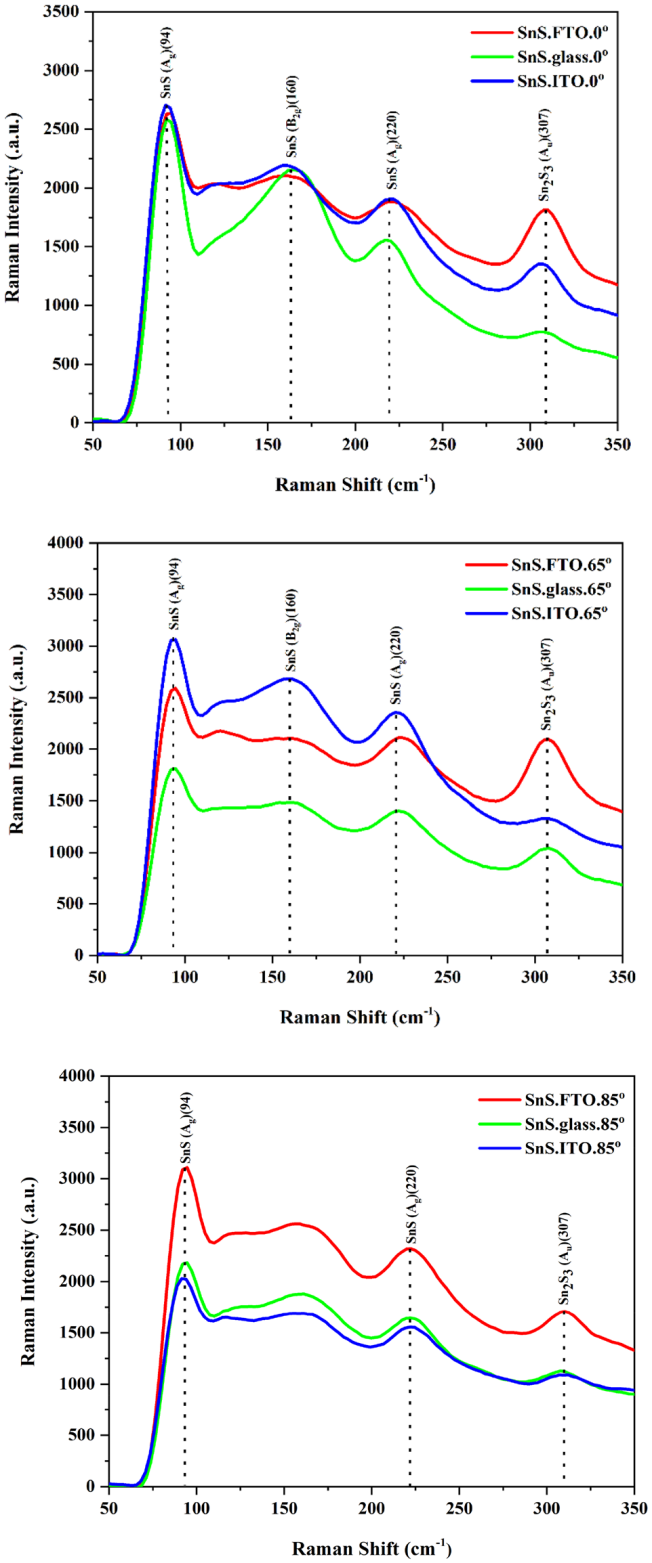
The Raman spectra of specimens produced at θ = 0°, 65°, and θ = 85° on (**a**) glass, (**b**) FTO, (**c**) ITO.

The Raman spectra of SnS nano-sheets at the deposition angles of 0°, 65°, and 85° on the glass, ITO, and FTO substrates have been illustrated in Fig. 3. SnS has 21 optical phonon modes; twelfth of which are active (4A_g_, 2B_g_, 2B), seventh of which are infrared active (3B_1u_, 1B_2u_, 3B_3u_), and two of which are inactive (2A_u_). The 94 and 218 peaks are related to the Transverse Optical (TO) mode of Ag^14^. One of the Raman peaks of the second phase of Sn_2_S_3_ is related to Ag mode that stems from the change in the atomic ratio of Sn to S^15^. As observed, due to the entanglement of phonon, the Raman peaks are emerged at low wavelengths^16^. Partial flattening is observed in the Raman spectra of some samples. The reason for this phenomenon is the strain between SnS and the substrates^17^. The noteworthy point is the change of the peak of Au (307) related to the second phase of Sn2S3. As it can be seen, with the change of the substrate at the same angle of deposition, this mode is the highest value for example with the FTO substrate and the lowest value for the glass substrate. In fact, it can be concluded that the substrate has a great influence on the formation of the second phase (Sn_2_S_3_). Mode control has also been observed in other materials. For example, Ben Cao and his colleagues observed that in the Raman spectrum, the intensity of the lnN peak gradually decreases, while the intensity of the ln_2_S_3_ peak increases with the increase of sulfurization time^18^. In addition to the lattice constant of the substrate, thermal mismatch between film and substrate is another parameter producing the strain of the original structure^19^. Both the substrate and the deposition angle cause the amorphization of the structure that leads to the flattening and the formation of the second phase of Sn_2_S_3_, which clearly results from the Raman analysis. As observed in Fig. 3, the slight dislocation in B2g mode is due to the restricted ordering introduced by grain boundaries in polycrystalline samples^20^. Compounds having similar structures such as GeS are very weak, which is also observable in the Raman modes of B_2g_^14^. The Raman analysis is a remarkable method for studying the structural properties of nanoparticles including their sizes^21^. The effect of nanoparticle size on the Raman spectrum has been extensively investigated by researchers. For example, Campbell and Fauchet have reported the dependence of the shift of the Raman peak on the particle size^22^. The same results have also been reported by the others^23–25^. Another effective parameter on the location of the Raman peak is the nature of the defect on the grain boundaries^26^. The change in the peaks’ position at higher wavelengths is a direct illustration of decrement in the particle size.

The phonons lifetime of films can be calculated by^27^:

2 $$\frac{1}{t} = \frac{{\Delta E}}{\hbar } = 2\pi c\Gamma \left( {10^{{ - 12}} \;{\text{s}}} \right)$$

where ΔE represents the uncertainty in the energy of the phonon mode, ћ denotes Planck’s constant divided by 2π and Γ is the full-width at half maximum of the Raman peak in units of cm^-1^. The related data of the phonon lifetime are illustrated in Table 2. As a result of changing the substrate type and the employed deposition angle, the crystalline structure of samples changes; one of which is the change in crystallite size that is effective in the phonons lifetime. Actually, with the increment of the crystallite size, the lifetime of phonon increases too^27^. In addition, with the increase of deposition angle and increase of the defects in the structure, the phonon lifetime increases^28^. As it is observed in Table 2, with the increase of deposition angle of the samples prepared on glass and ITO substrates, the phonons lifetime increases along with increment in the height of the main Raman peak. However, this is not the case for the samples deposited on FTO substrate. The remarkable emergence of Sn_2_S_3_ phase (increasing the crystallinity of Sn_2_S_3_) in FTO deposited samples may be the reason for the decrease of the phonons' lifetime. Especially, for the samples deposited on the FTO substrate at higher deposition angles, the same conclusion can be made; by which the crystallinity of the second phase (Sn_2_S_3_) remarkably improves and in turn the lifetime of the phonons decreases for the main Raman peak (94 cm^-1^).

**Table 2:** The phonon lifetime and Sn/S ratio of samples.

Sample (^o^)	FTO.0	FTO.65	FTO.85	glass.0	glass.65	glass.86	ITO.0	ITO.65	ITO.85
Sn/S	1.14	1.22	1.7	1.13	1.20	1.23	1.12	1.17	1.22
Phonon lifetime	0.9731	0.0692	0.1534	0.1683	0.3688	0.6913	0.2973	0.4001	0.6900

### EADX analysis

In order to study the effects of the applied technique and the substrate type on the final structure of samples, the EDAX analysis has been provided. With the increase of the deposition angle, the atomic ratio of atoms changes. This variation results in the formation of the second phase. The Sn/S ratio has been reported in Table 2.

One of the important factors that have a great impact on the changing the atomic ratio is the deposition method and increasing the thickness of the film. Mathur et al. studies variation of composition ratio in amorphous silicon carbide films. They were observed that with increasing film thickness, the atomic ratio of SiC also increases. The atomic ratio of silicon to carbon is also responsible for the shape, structural, electrical, and optical properties of the a-Si_1-x_C_x_ films^29^.

In deposited samples, the Sn/S atomic rate increases as the thickness of the nanoplates increases.

The most increase in the ratio is related to the SnS.FTO.85° sample. The atomic mass of S is more than that of Sn. With the increase of the deposition angle, maybe due to the further scattering and eject of S atoms from the surface of the substrate, the content of S decreases; leading to the formation of other phases of SnS.

The effect of the atomic ratio of the utilized materials has been the subject of many investigations. For example, Zhao et al., have deeply investigated the Si/Cu ratio changes and observed that the $\gamma$ values considerably changed based on the FESEM analysis^30^. For justifying this observation, they used the semi-experimental theory of the adjutant Si and Cu atoms by which the remarkable impact of the ratio of atoms was found^30^. Tanto et al.^31^ investigated the effect of Sn/S ratio on column angle evolution during oblique angle deposition.

### FESEM analysis

**Figure 4:**
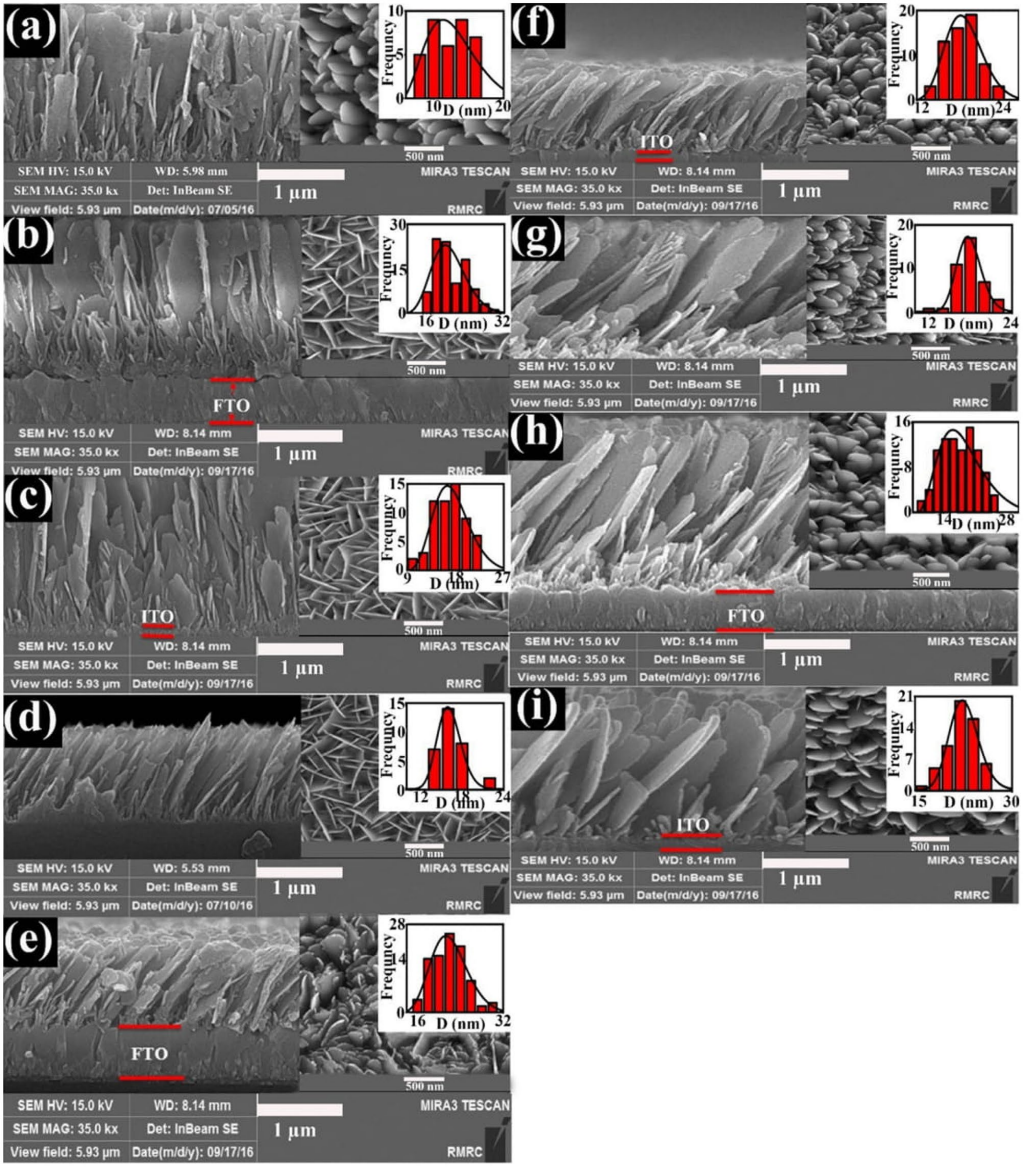
The FESEM cross-section image of (**a**) SnS.glass.0°, (**b**) SnS.FTO.0°, (**c**) SnS.ITO.0°, (**d**) SnS.glass.65°, (**e**) SnS.FTO.65°, (**f**) SnS.ITO.65°, (**g**) SnS.glass.85°, (**h**) SnS.FTO.85°, (**i**) SnS.ITO.85°, (The insets are the top view of the FESEM images and the particles size histograms).

**Figure 5:**
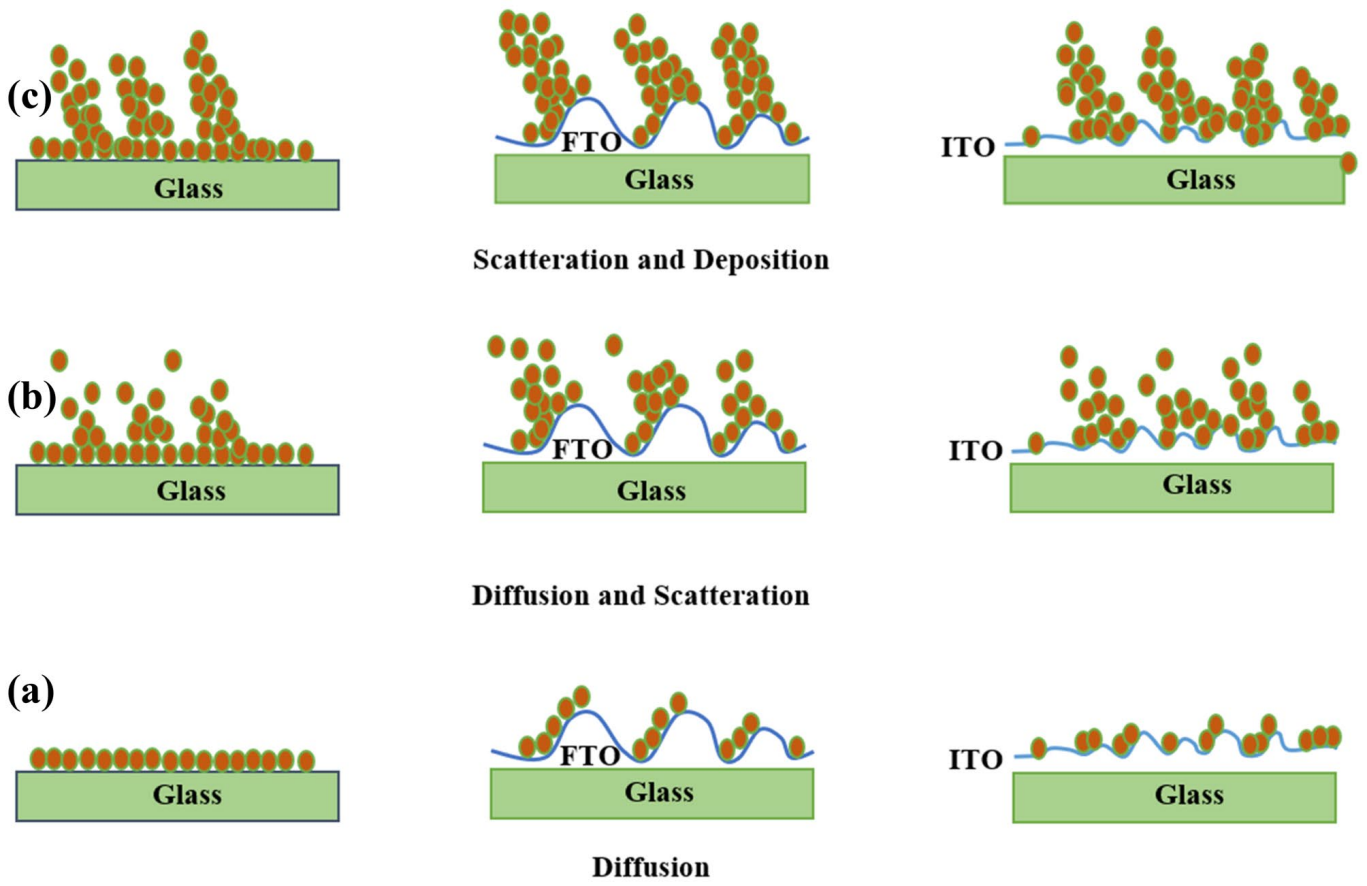
Schematic showing nano-sheet tin sulfide growth mechanism in three different stages (**a**) diffusion, (**b**) diffusion and scatteration, and (**c**) scatteration and deposition.

In Fig. 4, the FESEM image of SnS samples. Several factors result in the formation of SnS structure in other forms such as circular structure^32^, nanorods^33^, and nano-flakes^34^; some of which have been found to be dependent on deposition temperature, annealing^32^, change in Sn/S ratio^35^, and pollutants^8^. Park et al.^32^, have measured the growth temperature of the final morphology of samples. They observed the nano-sheets like growth behavior of SnS at room temperature and the change in structural properties with the increase of temperature. For instance, the porosity and in turn the density of samples increased, when the growth temperature was set to 280 °C. The annealing temperature is reported to be another effective factor in the growth process of the SnS layer. Ghosh et al.^36^, investigated the effect of substrate temperature on the final structure of the sample using the PVD technique. With the increase of the annealing temperature, the structure and morphology of the sample remarkably change from nano-sheets to other forms. However, at higher temperatures, this trend violates and the morphology of samples thoroughly changes. Variation in Sn/S ratio is another effective parameter on the morphology, which depends on temperature, and in Chemical Vapor Deposition (CVD) technique it depends on the density of materials^35,37^. Sb and Ge doping on SnS have been studied by Hsu et al.^38^. They found that the increase in the Sb and Ge dopant contents of SnS films changed their ultimate morphology compared to the un-doped one. The other notable observation is the change in the diameter of the nano-columns upon doping the SnS by In and changing the Sn/S ratio^39^. As observed in Table 1, with the increase of the deposition angle, the diameter of the grown nano-sheets increases. The formation of the second phase may be a reason for occurring this phenomenon. Besides that, according to Table 1, a direct relation is found between the diameter of the nano-sheets and the Sn/S ratio. The most remarkable change in the diameter is found for the SnS FTO.85 sample, for which the equivalent Sn/S ratio is 1.7. The suggested mechanism for the growth of nano-sheet like SnS film is shown in Fig. 5.

It is clear that different surfaces have different roughness. The incident flux coming from the source towards the substrate is conical, resulting in the non-homogeneous formation of the particles^3^. This non-homogeneity along with the existence of roughness leads to the emergence of noise on the substrate surface where the deposition process of the atoms begins. These deposited atoms can act as a barrier for the movement of the incident atoms, resulting in the formation of the primary SnS nuclei^40^. When atoms reach the substrate surface, they can move on the surface depending on the rate and the deposition angle but this movement is very low approximately between 0.1 and 0.2 eV and the deposition is ballistic. In the present case, after passing a distance on the surface, the atoms lose their energy immediately and the deposition process begins. After a while, due to the shadowing effect of atoms, nanoparticles do not grow in the middle of the nano-sheets that causes the increase of porosity^11,41^. When atoms perpendicularly collide with the initial nuclei, they may be reflected at various angles. This reflection depends on the primary energy; for example, for atoms with high energies, they are probable to be thoroughly reflected from the surface and hence the deposition process does not take place. For substrates with different roughness (FTO and ITO in this case), the existed seeds play the role of predetermined barriers, but the seeds of FTO and ITO substrates are not regularly distributed on the surface. These seeds have a determinative role in the porosity degree of samples^42^. In fact, the porosity depends on the location of these seeds. Hence, apart from the deposition angle, the effect of the substrate on the porosity of the samples is remarkable. Table 1 shows that the porosity values for samples deposited on FTO substrate are higher than those deposited on glass and ITO substrates. This effect has been investigated in the AFM section.

When substrates are situated at a certain angle, with respect to the source normal, and the particles reach the substrate, the nominal angle ($\alpha$) differs from the actual angle ($\gamma$) observed in FESEM images. Several approaches have been proposed for calculating this deviation. Tait et al.^43^, have proposed a geometrical approach without considering the properties of utilized material. Tanto et al.^31^, considering a relationship between the angles of the grown nano-sheets, have calculated the deposition angle of the incident flux. In this approach, the nature of the grown nano-sheets depends on the type of material and other conditions of the growth process such as pressure, substrate temperature, and the deposition angle of the incident flux.

The related angle of nano-sheets (φ) is a different parameter from the angle of nano-columns that depends on the type of the deposited material and the rate of growth. The φ values have been obtained from the cross-section image of the samples.

For calculating the angle of the grown nano-sheets, the following formula is used^31^:

3 $$\begin{aligned} \gamma & = \alpha - arctan\;arctan\left[ {\frac{{sin\;sin\left( \varphi \right) - sin\;sin\left( {\varphi - 2\alpha } \right)}}{{cos\;cos\left( {\varphi - 2\alpha } \right) + cos\;cos\left( \varphi \right) + 2}}} \right]\;\;\;for\,\alpha \le \varphi \\ \gamma & = \alpha - \frac{\varphi }{2}\;\;\;for\,\alpha \ge \varphi \\ \end{aligned}$$

The above relation properly involves the effects of the utilized material. The substrate effect has also been investigated in a research, in which the remarkable impact of them on $\gamma$ values has been concluded^44^. The intervals among the nano-sheets depend on the intervals among the seeds (their $\gamma$ values) they originated from and is interpreted as porosity. The reason for the increase in the distance of nano-sheets from each other is the shadowing and re-emission effects^11^. These effects are deeply investigated in the AFM analysis. Using FESEM images with the increase of the deposition angle the growth of some nano-sheets is ceased^11^. In fact, with the increase of the deposition angle, the atoms grow in directions to have the least energy value^11,12,45^. This property is also in accordance with the XRD analysis^11^. The deposition angle and the substrate type have remarkable impacts on the structure and morphology; one of which is the percentage of the porosity in thin films. The ability to control this factor is of great significance. With the increase in the deposition angle, in approximately smooth surfaces, the porosity of the samples increases due to the shadowing effect^40,46^. This property is accompanied by the refractive index of samples resulting in the increase of efficiency of samples in solar cells based on simulations^47^. Generally speaking, porosity and deposition rate has direct impacts on the refractive index and thickness^48^. For calculating the porosity, a geometrical model has been proposed by Poxson et al.^48^:

4 $$P=\frac{\gamma .tan(\gamma )}{C+\gamma .tan(\gamma )}\times 100$$

where $C=\frac{\pi {V}_{material}}{2{A}_{cs}h}$, $\gamma$ is the angle of grown nano-sheets in the FESEM images, V_material_ is the equivalent nano volume of the shadow, and A_CS_ is the cross-section area of the nano-columns; all of which are obtained using the FESEM images. These values are tabulated in Table 1. According to this relation, the absence of porosity is expected for the sample deposited at 0 angle, which stems from this fact that this relationship based on a geometrical view, without considering the phenomenal conditions of growth. The reason for the existence of porosity in a sample is the surface distribution phenomenon^40^. These observations have been also reported for the SnS sample deposited by the PVD technique^49^.

### AFM analysis

**Figure 6:**
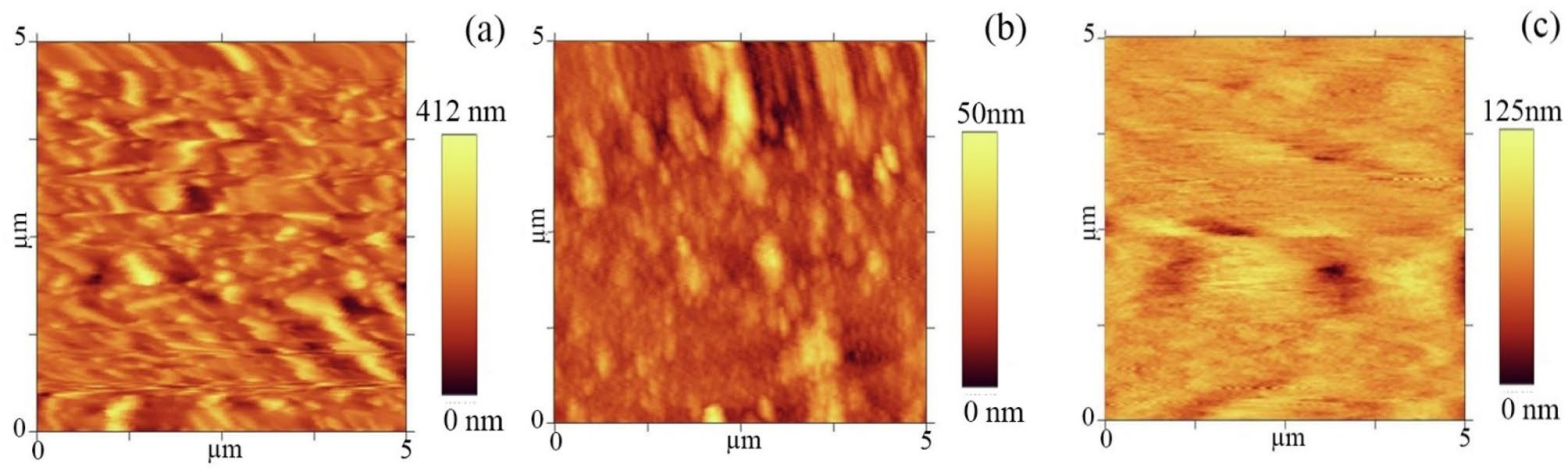
AFM images of (**a**) FTO, (**b**) glass, and (**c**) ITO substrates.

AFM analysis was used to fully investigate the effects of the substrate on the final structure. Figure 6 illustrates the surface section of FTO, glass, and ITO substrates, respectively. The average roughness of FTO, glass, and ITO is 46.88 nm, 12.17 nm, and 27.01 nm, respectively. As can be seen from the AFM images, the surface of the substrates has different morphologies. Considering the roughness values of the substrates, none of them is found completely smooth and ideal.

**Figure 7:**
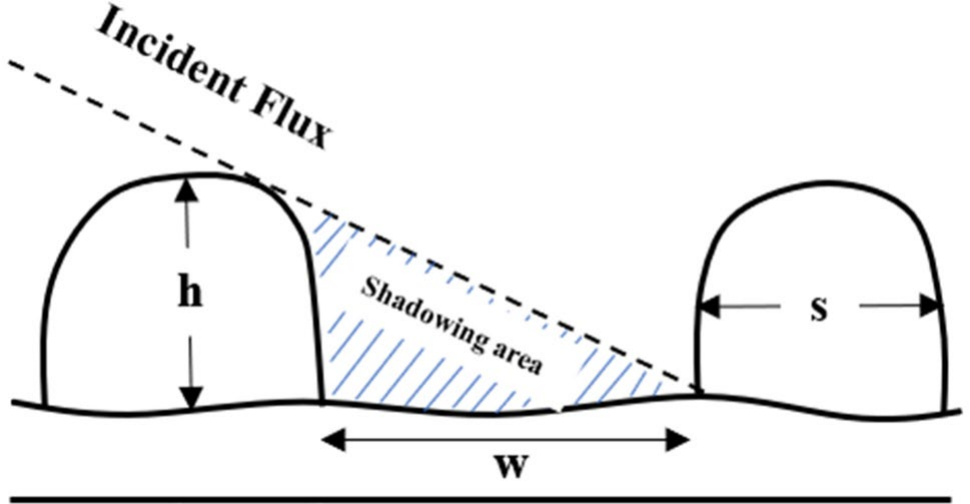
Schematic diagram of nano-growth of tin sulfide.

The glass substrate has lower roughness than FTO and ITO and hence has fewer seeds. The incident flux, therefore, does not homogenously reach the glass substrate, which leads to an increase of roughness^40^. Since the formation of nuclei is random on the substrate surface, the formation of nano-sheets is random too^50^. In this condition, the porosity is controlled by the deposition angle, since there is no remarkable number of seeds. Actually, the formation of nuclei is a result of the noise of the incident flux that the nano-sheets are formed on the existed seeds of ITO and FTO substrates. The existence of seeds on the substrate acting as the nucleation sites has remarkable effects on the final structure of the deposited films. At normal incident angle (i.e. θ = 0°) no sheets form behind the seeds and the growth of nano-sheets on the seeds, due to more volume of the incident flux, increases. This increase in the incident flux reaching the substrate surface results in the increase of the nano-sheets thickness. In addition, with the increase of the deposition angle, a greater number of the seeds are exposed to the deposited materials. Consequently, the thickness of sheets is affected by these conditions, which is provided in Table 1. With the increase of the deposition angle, the thickness of nano-sheets increases. This increase for the SnS.FTO.85° sample is found to be the most. The effect of the roughness of the substrate has been investigated by Whitacre et al.^44^. They found that the size of the shadow depends on the figure of the substrate, despite the reduction in the average flux of atoms on the surface. By comparing different types of substrates, it is inferred that the height of seeds on the substrate and deposition angle are the two parameters affecting the amount of adsorbed atoms resulting in the initiation of the nano-sheets growth. As observed in Table 1, for the samples deposited on FTO substrate, the maximum thickness of nano-sheets has resulted. Some specifications such as seed height, seed intervals, and the deposition angle have a direct impact on the porosity^42^. These factors have been illustrated in Fig. 7.

The porosity of the films deposited on substrates having seeds with equal intervals is controllable, and it reaches its maximum value on condition that the nano-sheets grow on the seeds thoroughly. The relation between the seeds’ interval and height, and the deposition angle is as follows^42^:

5 $$S\le tantan \left(\alpha \right) .h$$

where ‘S’ and ‘h’ are, respectively, the height and the seeds interval. In an ideal condition, the growth of nano-sheets thoroughly happens on the seeds; otherwise, nano-sheets grow among the seeds. This shows that the porosity generally depends on seeds specifications and deposition angle. The above relation is essential for the growth of periodic structures that have substrates with regular seeds^40^. The notable point is that in some samples at the deposition angle of α and in equal intervals of seeds, some of the nano-sheets are not formed on the seeds. The reason for this phenomenon is well understood if the geometry of the incident flux reaching the substrate surface is considered as a cone, with the deviation from the center of the of cone, the flux density can be less or more^51^.

## Conclusion

The SnS samples were successfully deposited on glass, ITO and FTO substrates at 0, 65 and 85° angles by GLAD technique. Various factors such as strain from the substrate, the formation of the second phase of Sn2S3 had an effective effect on the structural and morphological properties of SnS layers. The substrate type, depending on its nature (amorphous or lattice) along with its physical properties such as height and distance of the sides from each other has a great impact on the morphology and porosity of the samples. On the other hand, the deposition angle as a controllable factor causes the sample to amorphize at high angles and the result is the formation of the second phase of Sn_2_S_3_ which was observed in XRD and RAMAN analysis. Porosity in the samples affected by the substrate and the deposition angle were well observed in FESEM and AFM analysis. As the deposition angle increased and the samples became amorphous, EDAX analysis showed that the Sn to S ratio changed, resulting in the formation of the second phase. Increasing the thickness of the nano-sheets, flattening in the RAMAN spectrum is one of the effects of the second phase. At the end, the growth of the layers was explained and from the FESEM images, the growth angle of the samples was analyzed.

## Data Availability

The datasets used and analyzed during the current study available from the corresponding author on reasonable request**.**
